# Twenty-five years of Medical Library Association competencies and communities

**DOI:** 10.5195/jmla.2024.1966

**Published:** 2024-07-01

**Authors:** Stephanie Fulton, Gale G. Hannigan, Rikke S. Ogawa, Jodi L. Philbrick

**Affiliations:** 1 s-fulton@library.tamu.edu, Associate University Librarian for Health Sciences & Veterinary Medicine, University Libraries, Texas A&M University, College Station, TX; 2 Former Research Professor/Research Services Librarian, Health Sciences Library and Informatics Center, University of New Mexico Health Sciences Center, Albuquerque, NM; 3 rsogawa@uci.edu, Assistant University Librarian for Public Services, UCI Libraries, University of California Irvine, Irvine, CA; 4 Jodi.Philbrick@unt.edu, Principal Lecturer, Department of Information Science, University of North Texas, Denton, TX

**Keywords:** MLA competencies, Health Information Professionals, Medical Library Association, Organizational Change

## Abstract

Professional associations provide resources to support members' career development and facilitate ways for members to engage with and learn from one another. This article describes Medical Library Association (MLA) activities related to the revision of professional competencies and the restructuring of the organization's communities during the past twenty-five years. Grounded in MLA's Platform for Change, the MLA competency statement underwent two revisions with core themes remaining consistent. Major efforts went into rethinking the structure of MLA communities, and it became a strategic goal of the association. Numerous groups spent considerable time guiding the changes in MLA's community structure. Sections and special interest groups were transformed into caucuses. Domain hubs were established to facilitate project coordination across caucuses and create more leadership opportunities for MLA members, but their implementation did not meet expectations. Member engagement and leadership are ongoing challenges for MLA. The next twenty-five years will undoubtedly see additional revisions to the competencies and continued iterations of the community structure.

## INTRODUCTION

The 1990s saw widespread use of the World Wide Web, personal digital assistants (PDAs), and email to name a few technologies that allowed people to find and communicate information. Digitized library collections were new, as was Google, enabling easier access to information outside of the physical library. PubMed was launched in 1997, and electronic medical records systems were being developed, making biomedical and patient information more available. By the time the Medical Library Association (MLA) celebrated its 100th anniversary in 1998, it was apparent that health professionals' learning had to be fast-paced and continuous. Continuing education (CE) courses at the 1998 meeting included: Managing infoGLUT: Managing Too Much Electronic Information, Building and Managing Your Digital Library, Basic Web Page Design, and Consumer Health Information on the Internet. The MLA 1999 Annual Meeting was aptly themed “Present Tense, Future Perfect?” Since then, we have witnessed ongoing and significant changes in the information landscape – think Web 2.0, social media, smart phones, big data, rapidly emerging generative artificial intelligence (AI). Advances in molecular medicine, including genetic analyses, provide increased understanding of disease processes, enabling the development of more “personalized” medicine. Improved imaging technologies give 3D views of a patient's unique anatomy and physiology and can be invaluable in guiding surgical procedures. Many of these advances involve large sets of data. Changes in the health care landscape naturally influence the necessary knowledge and skills we need as health information professionals.

Professional association activities, such as those established and supported by MLA, play a key role in ensuring their membership has the knowledge and skills to practice effectively now and in the future. In this article, we focus on MLA's professional competencies and the development of MLA communities. Defined professional competencies inform and guide the professional development activities of practitioners; MLA communities connect members with similar interests. MLA's competencies emphasize an individual's responsibility to seek opportunities to learn continuously and acquire skills needed as roles change. Competencies assist in reflection and self-assessment; professional communities facilitate communication among practitioners with similar roles and interests. These are aspects of more informal professional development. Changes in MLA's support for more structured, formal professional development opportunities, such as MEDLIB-ED and specialization certification, are discussed elsewhere in this issue.

The perspective shared in this article is from MLA members who participated in the development of MLA's 2017 *Competencies for Lifelong Learning and Success* (Hannigan and Philbrick) and different phases in the restructuring of MLA communities (Fulton, Ogawa, and Philbrick). Our purpose is to highlight the changes in the competencies for health sciences librarians and in the communities (focusing on the former Section Council, which was comprised of representatives from sections and special interest groups) within MLA over the past 25 years. The processes in making these changes were not symbiotic, but the changes to the competencies did inform the process for changing the community structure within MLA. We acknowledge that the competencies and community structure will not remain static over time, with the processes to review and refine them being iterative and evolving.

## EVOLUTION OF THE MLA PROFESSIONAL COMPETENCIES

At MLA's Centennial, Fred Roper reflected on MLA's longstanding commitment to professional development. He quoted Louise Darling, who said, on the occasion of MLA's 75th anniversary, “our Association has been talking about education, standards, and certification for most of its life.”[1] *Platform for Change: the Educational Policy Statement of the Medical Library Association* responded to the “need for a clear and forward-looking statement of expectations for medical librarians and…an agenda for future action.”[2] This report addressed the continuum of learning to support a medical librarian's competence and performance, including formal training and more informal opportunities. The authors identified graduate programs, MLA and other professional associations, the National Library of Medicine (NLM), employers, vendors, and publishers as sources of training, but stated that “the ultimate responsibility for lifelong learning and professional development rests with the individual.”[2]

In 2007, the MLA Task Force on Educational Policy Statement Revision reviewed *Platform for Change*, acknowledging the dramatic change in the health and biosciences environment and the ubiquitous role of technology. Member input had been solicited at the MLA 2005 Annual Meeting, and the overall goal was to develop “an overall strategic statement of MLA's approach to education and professional development for its members in the future.”[3] The Task Force released a new educational policy statement, *Competencies for Lifelong Learning and Professional Success: The Educational Policy Statement of the Medical Library Association*, identifying seven professional competency areas [3].

As in *Platform for Change*, the authors emphasized the individual's responsibility for professional development and the necessary support roles of MLA, employers, educators, and NLM. For example, recommendations for MLA indicated that “MLA must continue its leadership role in creating a vital and responsive professional development program and a dynamic set of coordinated education opportunities.”[3] The scope of this report also went beyond competencies to include personal attributes such as “political savvy and negotiation acumen,” “adaptability and flexibility,” and “balance of personal and professional life.”

Competency statements need to be updated to reflect the continuously changing nature of the health information environment and practitioners' roles. In 2017, MLA's Task Force to Review MLA's Competencies for Lifelong Learning and Professional Success issued its report including revised professional competencies [4]. During the time since the 2007 report, other professional organizations had issued competency statements, which informed the Task Force's work. The 2017 revision followed the practice that “competency statements define essential professional skills and abilities that can be observed, measured, and taught.”[4] The Task Force held an open forum at the MLA 2015 Annual Meeting and distributed a survey to the entire MLA membership to solicit input to inform the revision. Multiple drafts of competency statements were reviewed by early career and experienced professionals, members of other associations, and the MLA Research Imperative Task Force. A project award from the National Network of Libraries of Medicine (NN/LM) Southeastern/Atlantic Region supported the revision of the competencies by handpicked MLA leaders and experts. Because technology had become so pervasive in our work, reference to technology skills was woven throughout the document rather than appearing as a standalone competency. Research skills were also emphasized, as was the broader role of health information professionals as educators. The resulting *Medical Library Association Competencies for Lifelong Learning and Professional Success, 2017* lists six competencies:

- Competency 1, Information Services: A health information professional locates, evaluates, synthesizes, and delivers authoritative information in response to biomedical and health inquiries.
- Competency 2, Information Management: A health information professional curates and makes accessible bioscience, clinical, and health information data, information, and knowledge.
- Competency 3, Instruction & Instructional Design: A health information professional educates others in the skills of bioscience, clinical, and health information literacy.
- Competency 4, Leadership & Management: A health information professional manages personnel, time, budget, facilities, and technology and leads others to define and meet institutional goals.
- Competency 5, Evidence-Based Practice & Research: A health information professional evaluates research studies, uses research to improve practice, conducts research, and communicates research results.
- Competency 6, Health Information Professionalism: A health information professional promotes the development of the health information professions and collaborates with other professionals to improve health care and access to health care information.

Structurally, each competency statement is followed by an explanation, performance indicators, and examples of basic and expert levels of performance. For example, Competency 2 addresses information management. The explanation, one of the performance indicators, and examples at both the basic and expert levels are given here:

- **Competency:** Competency 2, Information Management: A health information professional curates and makes accessible bioscience, clinical, and health information data, information, and knowledge.
- **Explanation:** Our strength is our ability to develop and organize collections tailored to specific audiences. In cataloging and classifying, including assigning metadata, we impose order to improve access. Traditionally, we have organized information resources into libraries, and personal records and artifacts into archives. Now, our expertise is being applied to organizing research data into collections that can be used electronically across institutions and countries. We know the value of and how to apply standards so that records of collections are universally comprehensible and enduring.
- **Example Performance Indicator:** Implements data management plans.
  - **Basic:** Describes the data life cycle; identifies and describes data resources, tools, and repositories; explains data plan requirements of funding agencies.
  - **Expert:** Conducts data curation interviews; develops and implements data management plans and policies; consults on managing data across the data life cycle.

The three versions of MLA's professional competency statements are mapped in Table 1. Six common competency themes are found among the three, including information services, information management, instruction, management, research, and technology. Technology only appears in the 1992 and 2007 iterations, as technology was woven throughout the 2017 statement.

**Table 1:** Mapping of MLA competency statements

Competency Theme	1992	2007	2017
Information Services	**Health Sciences Information Services:** “Health sciences librarians require knowledge of the content of information resources and skills in using them”	“Understand the principles and practices related to providing **information services** to meet users' needs”	**Information Services:** “A health information professional locates, evaluates, synthesizes, and delivers authoritative information in response to biomedical and health inquiries”
Information Management	**Health Sciences Resource Management:** “Health sciences librarians must know the theory of, as well as have skills in, identifying, collecting, evaluating, and organizing resources and developing and providing databases”	“Have the ability to **manage health information resources** in a broad range of formats”	**Information Management:** “A health information professional curates and makes accessible bioscience, clinical, and health information data, information, and knowledge”
Instruction	**Instructiona 1 Support Systems:** “Teaching ways to access, organize, and use information to solve problems is an essential and everwidening responsibility of the health sciences librarian”	“Understand **curricular design and instruction** and have the ability to teach ways to access, organize and use information”	**Instruction & Instructional Design:** “A health information professional educates others in the skills of bioscience, clinical, and health information literacy”
Management	**Management of Information Services:** “Leadership in the application of library and information science to the handling of health sciences information resources in complex institutional environments”	“Know and understand the **application of leadership, finance, communication, and management** theory and techniques”	**Leadership & Management:** “A health information professional manages personnel, time, budget, facilities, and technology and leads others to define and meet institutional goals”
Research	**Research, Analysis, and Interpretation:** “[T]he health sciences librarian is called upon to apply knowledge, skills, and understanding” to conduct and interpret research	“Understand **scientific research methods** and have the ability to critically examine and filter research literature from many related disciplines”	**Evidence-Based Practice & Research:** “A health information professional evaluates research studies, uses research to improve practice, conducts research, and communicates research results”
Health Information Environments/Profession	**Health Sciences Environment and Information Policies:** “Health sciences librarians must understand the contexts in which the need for biomedical and related information emerges and the unique ways of perceiving and interpreting those environments”	“Understand the **health sciences and health care environment** and the policies, issues, and trends that impact that environment”	**Health Information Professionalism:** “A health information professional promotes the development of the health information professions and collaborates with other professionals to improve health care and access to health care information”
Technology	**Information Systems and Technology:** “Health sciences librarians must be able to understand and use technology and systems to manage all forms of information”	“Understand and use **technology and systems** to manage all forms of information”	Note: Not included as a standalone competency, but rather woven throughout the competencies listed above.

Over time, researchers have written about various aspects of competencies as they relate to health sciences librarians; the following are some examples.

In 2012, Philbrick conducted a Delphi study “to identify the professional and personal competencies that entry-level academic health sciences librarians should possess from the perspectives of academic health sciences library directors, library and information sciences (LIS) educators who specialize in educating health sciences librarians, and individuals who serve as both LIS adjunct faculty and practitioners in the field of health sciences librarianship.”[5] She found that, for the entry-level academic health sciences librarian, personal competencies are as important as professional competencies. Academic health sciences library directors emphasized the importance of teamwork, learning, integrity, motivation, flexibility, and communication [5].

Ma, Stahl, and Knotts conducted a scoping review to identify emerging roles of health sciences information professionals (HIP) to “inform library school students about expected entry-level job qualifications and faculty about adaptable changes to specialized HIP curricula.”[6] Nine categories of roles were identified, such as clinical and medical information provision, data management, research, and scholarly publishing. All emerging roles involved multiple MLA professional competencies.

Bass et al. focused on the competencies needed to develop skills in collection organization [7]. They reviewed formal and informal opportunities to develop those skills and found that communities of practice are important resources for people involved with cataloging and metadata. They advocated for the need for more formal and informal opportunities for librarians to develop and grow these skills.

The MLA professional competencies, along with researchers' work in this area, have outlined the knowledge and skills required of health sciences librarians, and there is a continual need for health sciences librarians to stay current on the latest developments in the field. MLA's community structure has provided a framework for MLA members to learn from each other, and its evolution is discussed in the subsequent sections.

## EVOLUTION OF THE MLA COMMUNITY STRUCTURE

In June 1977, the MLA membership accepted the recommendations of the Ad Hoc Committee to Study MLA Group Structure, leading to the development of sections and Section Council [8]. Starting in 1980–81, the main community structure of MLA consisted of sections (groups whose members paid dues to support the activities of the section and consisted of a specified number of members set by MLA) and special interest groups (SIGs, groups whose coalesce around emerging areas of interest, did not necessary have enough members or routinized leadership to be developed into a Section, and whose membership didn't require due), which fell under the umbrella of Section Council [9] (the governing body made up of elected representatives, whose task included approving or sunsetting the formation of Sections and SIGs, program planning, and supporting MLA initiatives). The following years allowed for examination of these community structure changes made in the early 1980s. There have been several groups who have examined those changes and made recommendations and/or took actions regarding MLA community structure over the last 25 years, as outlined in Table 2.

**Table 2:** Timeline of MLA community structure recommendations/actions (1998-present)

Years	MLA Group	Relevant Recommendations/Actions
1999	Governance Task Force [10]	Reduce the size of Section Council by eliminating the underutilized position of alternate Implement a new structure of Section Council based on a representative-elect model Result: Implemented
2007	Section Council Composition Task Force [11]	Continue existence of Section Council and have it be composed of section chairs and immediate past section chairs instead of representatives from each section Result: Implemented
2009	Section Council Review of Section Pro gr amming Task Force [12]	Change from section-led programming for the annual meeting to a more general call for papers through Section Council Result: Implemented
2013–2014	MLA Futures Task Force [13]	Establish domains to define MLA's scope Streamline the organizational structure of MLA Result: Not implemented, but informed future task forces' work
2015–2016	MLA Strategic Priorities Task Force [14]	Reviewed section and SIG data for relevancy Developed relevance matrix for sections and SIGs Revised definitions of sections and SIGs
2016–2017	Rising Stars Cohort [15]	Create a metrics dashboard and standardized metrics form for Section Council Develop an automated system for sections to align their goals with MLA's strategic plan Improve visibility of sections' and SIGs' work on MLA's website Create a “SIG only” MLA membership with a nominal fee Encourage SIGs to host more activities online Result: Implemented system for aligning goals; Informed further MLA Board discussions and future task forces' work
2016–2019	Communities Strategic Goal Task Force [16]	Change sections and SIGs to caucuses (affinity groups in the document), Section Council changed to Community Council. Provide structure for communities to work together and connect with MLA programming/committees through communities of practice (eventually called domain hubs) Eliminate membership dues for sections to provide more access to participation Support the work of communities and hubs through MLA's annual budget Result: Recommendations received by Board, used to inform subsequent changes to bylaws, and served as outline for Communities Transition Team work.
2019–2020	Communities Transition Team [17]	Established caucuses Created policies governing caucuses, domain hubs, and Community Council Coordinated new budget model for communities with MLA Finance Committee Result: New community organizational structure established for MLA
2022–2023	Community Assessment Team [18]	Disband the domain hubs Result: Domain hubs disbanded May 2024.

In May 2016, the MLA Board of Directors approved a strategic goal focused on communities, which was intended to “[s]trengthen MLA's member communities (sections and SIGs) by analyzing and recommending community architecture and roles….”[19] This action was taken in response to the recommendations and suggestions from three groups (Futures Task Force, Strategic Priorities Task Force, and 2016–2017 Rising Stars cohort) that sections, SIGs, and/or Section Council needed to change. The MLA Communities Strategic Goal Task Force (CSGTF) was charged with completing the work to fulfill the strategic goal.

CSGTF began their work with a review of the previous groups' reports. At the MLA 2017 Annual Meeting, CSGTF engaged Section Council in a discussion about the ideal roles of sections, SIGs, and Section Council and whether the current organizational structures enabled these roles. Section Council members unanimously agreed the current structure did not enable the ideal roles. Together, CSGTF and Section Council began developing an initial set of guiding principles for an effective community [20]. The guiding principles and consensus of Section Council were also presented to MLA members at an open forum on the last day of the MLA 2017 Annual Meeting and distributed through MLA-FOCUS. It was clear in May 2017 that MLA needed to reconsider what structure would be best to support its members in engaging in communities and plan for a successful future.

Areas of concern that had been identified in previous reports, and repeated frequently through CSGTF's background work, included:

- Unequal access to support community engagement in and financial support for programming
- Difficulty engaging members in leadership opportunities, while at the same time, members felt it difficult to break into service at the national level (communities are national service opportunities)
- Difficulty working across sections to take action at a grassroots level
- Visibility of sections' and SIGs' contributions

The benefits envisioned for community membership included providing opportunities for people to coalesce around topics of mutual interest, share ideas, and improve professional work by engaging in meaningful activities.

Section Council was a multi-tiered system within MLA. If a section had a significant number of members (and, therefore, a significant treasury), the section could provide many benefits to their members (e.g., scholarships, meals, multiple programs at the annual meeting, etc.). Because SIGs had none of these resources, they depended on sections to partner with them for access to programming times at the annual meeting. If a section had a strong leadership tradition, leaders were coached on how to work collaboratively with other sections, how to engage with headquarters, and how to accomplish their goals. Sections with less stable leadership and SIGs, due to their structure, did not have that built-in support. At the MLA 2017 Annual Meeting, CSGTF heard from Section Council and MLA members that the organization needed a system that engaged members and enabled their interdisciplinary work to advance the organization's core areas of interest.

The following association year (2017–2018), CSGTF debated proposed frameworks for a renewed community structure. The task force referred to the guiding principles, feedback received at the MLA 2017 Annual Meeting, recommendations from previous groups, and member input received during the brainstorming and design phase. CSGTF coalesced around a structure originally called Communities of Practice, later called Domain Hubs. Each affinity group/caucus (formerly a section or SIG) would align with at least one community of practice/domain hub. The domain hubs, an idea that grew out of work from previous task forces, aligned the caucuses' work with the MLA professional competencies and the practice areas of Clinical Support, Education, Health Equity & Global Health, Information Management, Information Services, Innovation & Research Practice, and Professionalism & Leadership [4,13]. A domain hub would be a supporting group to facilitate the work across caucuses and would be connected to MLA programs and committees to provide support as well as recommendations for activities and leaders. To signify the new structure, Section Council would become Community Council representing the equal footing of all caucuses. As part of this framework, section dues would be eliminated. Any MLA member would be able to join any number of caucuses without financial barriers. Furthermore, the work of caucuses and hubs would be supported by the MLA budget.

CSGTF solicited feedback from section chairs (current and incoming), SIG conveners (current and incoming), and MLA's Diversity and Inclusion Task Force during the design phase, January - April 2018. Prior to the MLA 2018 Annual Meeting, CSGTF provided MLA membership with the proposed framework. Members of CSGTF offered every section and SIG an opportunity to have a task force member come to their annual meeting (in person or virtually) to discuss the framework, ask questions, and address concerns. Task force members attended 43 meetings of sections/SIGs; held a well-attended open forum at the MLA 2018 Annual Meeting in Atlanta, Georgia; and hosted two additional virtual meetings to provide space for members' input. After incorporating feedback received from various stakeholders, CSGTF presented the proposed framework to the MLA Board of Directors, who approved the framework at a meeting in summer 2018 [16]. A new group was then formed and charged with the implementation of the new framework: the Community Transition Team.

## COMMUNITY TRANSITION TEAM

Building on the work and recommendations from previous task forces, the Community Transition Team (CTT) was established in November 2018. An ambitious goal was set to have all sections (22) and SIGS (26) become caucuses by September 1, 2019.

Working groups were established to address key aspects of the new structure:

- Domain Hub Startup Workgroups
- Community Policies Workgroup
- MLA Committees Workgroup
- Finance Workgroup

It was through the work of the volunteers and MLA staff in these working groups that the overall goal of changing the MLA community structure was achieved. Details on the mission, deliverables, and timelines for each of these working groups is available on MLANET [21].

### Domain Hub Startup Workgroups

Seven Domain Hub Startup Workgroups, each with four members, did an incredible amount of planning and development to bring structure to this new element of MLA communities. Domain hubs were established to facilitate project coordination across caucuses and create more leadership opportunities for MLA members. Visions and first year milestones were established by every domain hub.

### Community Policies Workgroup

The Community Policies Workgroup created new policies to guide the structure and responsibilities of caucuses, replacing section bylaws and manuals. The resulting document, *Medical Library Association Board of Directors Policies Governing Caucuses, Domain Hubs, and Community Council*, was approved on July 22, 2019, by the MLA Board of Directors [22]. Working with the MLA Bylaws Committee, it was determined that sections becoming caucuses was a nomenclature change rather than a functional one. A vote of MLA membership would not be required to change to the structure of MLA sections, sigs, or section council into the proposed causes, domain hubs, and community council.

### MLA Committees Workgroup

The Committees Workgroup reviewed existing committees and made recommendations for updates and changes, which were approved at the November 2019 MLA Board of Directors meeting. A significant change was to ensure that there were formalized liaisons from the seven domain hubs to MLA committees. Previously, committee or jury appointments may have liaisons to related areas (e.g., Oral History committee often had a liaison from the History of Health Sciences section) but it was not required. This change included liaisons to the editors of *MLA Connect* and the *Journal of the Medical Library Association (JMLA)*; members of the National Program Committee; and members of the Education Curriculum Committee. Finally, revisions were made to the Academy of Health Information Professionals (AHIP) point index to include five new roles related to communities. References to the caucuses and communities were included in the MLA Bylaws adopted in 2023.

### Finance Workgroup

The Finance Workgroup was charged with defining the 2020 budget for community initiatives and the use or reallocation of accumulated section funds. Sections were legally part of MLA and not separate entities, so the MLA Board of Directors had ultimate authority and responsibility for the management of these assets [23]. Section treasurers were included in discussions and made recommendations to the Board of Directors for the use of their existing funds. A process was developed for domain hubs and caucuses to request funding for projects.

In addition to the operational changes and logistics needed to transform sections and SIGs into caucuses, communication with MLA members was understood to be an important element for success. Open forums were held at the MLA 2019 Annual Meeting to provide updates and answer questions; several posts to *MLA Connect* were written by leaders of CTT and other members of the various working groups; and interviews with MLA community leaders were conducted and posted on the MLA website.

**Figure 1:**
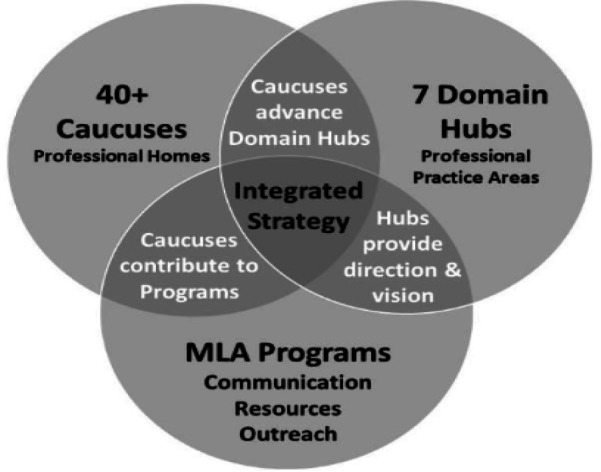
Collaboration, content, programming, and communication matrix [24].

Figure 1 was a central communication element used in presentations and on the MLA website to illustrate the connections within the evolving MLA organizational structure. The text in white describes the intended synergies from the new structure.

## IMPACT OF THE NEW COMMUNITY STRUCTURE

Reviewing the outcomes of the transition to the caucus and domain hub structure, there have been both successes and failures. Houk et al conducted an early assessment of the change process with recommendations for communication and trauma-informed practices for further changes [24]. Focusing on the goals originally set forth in the charge, the removal of financial barriers to joining a caucus led to an overall growth in the number of individual members joining caucuses and the number of caucuses a member joined. In 2018, the ratio of section members to MLA members was 2:1. By 2023, the ratio of caucus members to MLA members had grown to 4.4:1 (see Table 3). Along with the opportunity to join groups without financial barriers, the structure provided more opportunities for leadership growth by creating domain hub representatives (a member of a caucus who would represent that caucus's interests in discussions of domain projects/efforts) and domain hub chairs. Furthermore, the Community Council manual required that these positions be filled by different members (no member could serve in two or more caucus leadership roles) to create opportunities for member engagement. Unfortunately, with the uncertainty of the first year of the new structure, it was difficult to find members willing to step into these roles.

**Table 3:** MLA and community membership. 2018–2023

	2018	2019	2020	2021	2022	2023
**MLA Members**	2,663	2,348	2,341	2,370	2,370	2,389
**Caucus Members**	5,310	5,838	7,279	8,729	8,634	10,559
**Sections**	22	21	n/a	n/a	n/a	n/a
**SIGs**	26	27	n/a	n/a	n/a	n/a
**Caucuses**	n/a	n/a	43	43	43	42

This brings us to early 2020. During the first full year of implementation of the new structure for communities, the world experienced a public health crisis, the COVID-19 pandemic. The best-laid plans for continuing engagement with association members were quickly falling by the wayside as MLA began to realize that an in-person annual meeting, which was helpful to previous task forces in sustaining momentum, may not happen. While a virtual annual meeting was held in mid-summer 2020, the emotional labor required by all members to sustain the change process rightly was realigned to work supporting health care professionals. Our individual responsibilities of protecting the welfare of our communities, caring for family members, and caring for our own physical and mental health became paramount. The CTT was designed to complete the transition of MLA's organizational structure in 2020–2021, a season of completely remote activities, significant repeated public health crises, and a growing racial justice movement in the United States. The caucus plus domain hub structural transition of MLA communities did not evolve as originally planned because changes to organizational structure became a lower priority.

Recommendations for assessing these changes to organizational structure originated with CSGTF. These ideas were shared by the CTT with the MLA Board of Directors, and a further Ad Hoc committee was established in 2023 to assess the effectiveness of the changes [18]. With additional input and modification, surveys and interviews of MLA members were conducted. It was determined that, in most cases, the domain hubs did not facilitate structured collaboration as had been hoped and the additional leadership opportunities created proved challenging to maintain. A recommendation to sunset domain hubs was approved by the MLA Board of Directors in August 2023. Domain Hubs remained active until May 31, 2024.

## CONCLUSIONS

MLA is the professional home for many health science information professionals and has continued to evolve to better support their professional development and networking needs. In doing so, MLA must also remain a sustainable and relevant association.

Formal competency statements help define professional practice and guide professional development activities. MLA's professional competencies fall within broad themes that endure over time, but specific, measurable performance indicators do change. These performance indicators help an individual determine levels of personal proficiency. The MLA Competencies Self-Assessment tool is an aid for determining what skills a person might want to acquire or improve in proficiency as their job titles and roles change [25]. An individual is not expected to achieve mastery in all competency categories.

MLA's professional competencies have influenced the content of its educational programs, discussed elsewhere in this issue. In addition, MLA's competency statements have provided a framework for medical librarianship courses in graduate programs and helped set expectations for information professionals new to the field. Competency statements require ongoing review and revision; they are not static. The MLA competencies are due for review and revision in the next two years. These competencies have also guided the evolution of MLA communities, and competencies play an active role in MLA member and caucus goal setting.

MLA's organizational structure of communities has been redesigned over the years but continues to be a challenge.

In a member-driven organization, structural changes can only do so much to increase engagement. Members drive the priorities and opportunities for our own future. In the next 25 years, the authors look forward to the continuing efforts to promote member involvement in a thriving MLA.

## IN MEMORY OF GALE G. HANNIGAN

Stephanie, Rikke, and Jodi would like to take a moment to remember Dr. Gale G. Hannigan and acknowledge her work and years of service to MLA and librarianship. Without her wisdom and persistence, the 2017 revision of the MLA Competencies and this article would not have come to fruition. We were moved by her professional excellence and perseverance and feel fortunate to have worked with her on this project. Her beautiful spirit lives on in glimpses of the natural world.
